# Novel Therapeutic Approach for Obesity: Seaweeds as an Alternative Medicine with the Latest Conventional Therapy

**DOI:** 10.3390/medsci12040055

**Published:** 2024-10-13

**Authors:** Rajesh Yadav, Ankita Nigam, Richa Mishra, Saurabh Gupta, Anis Ahmad Chaudhary, Salah-Ud-Din Khan, Eman Abdullah almuqri, Zakir Hassain Ahmed, Sarvesh Rustagi, Deependra Pratap Singh, Sanjay Kumar

**Affiliations:** 1 Department of Dialysis Technology, Sharda School of Allied Health Science, Sharda University, Greater Noida 201310, Uttar Pradesh, India; 2 Department of Physiology, All India Institute of Medical Science, New Delhi 110029, India; 3 Department of Physiotherapy, Sharda School of Allied Health Science, Sharda University, Greater Noida 201310, Uttar Pradesh, India; 4 Department of Computer Engineering, Parul Institute of Engineering and Technology (PIET), Parul University, Ta. Waghodia, Vadodara 391760, Gujarat, India; 5 Department of Biotechnology, GLA University, Mathura 281406, Uttar Pradesh, India; 6 Department of Biology, College of Science, Imam Mohammad Ibn Saud Islamic University (IMSIU), Riyadh 11623, Saudi Arabia; 7 Department of Biochemistry, College of Medicine, Imam Mohammad Ibn Saud Islamic University (IMSIU), Riyadh 11623, Saudi Arabia; 8 Department of Mathematics and Statistics, College of Science, Imam Mohammad Ibn Saud Islamic University (IMSIU), Riyadh 11632, Saudi Arabia; 9 Department of Food Technology, School of Applied and Life Science, Uttaranchal University, Dehradun 248007, Uttarakhand, India; 10 Department of Biotechnology, Graphic Era (Deemed to be University), Dehradun 248002, Uttarakhand, India; 11 Department of Life Sciences, Sharda School of Basic Sciences and Research, Sharda University, Greater Noida 201310, Uttar Pradesh, India

**Keywords:** seaweed, brown algae, inflammation, obesity, therapeutic potential

## Abstract

The prevalence of overweight and obesity is increasing worldwide. Common comorbidities related to obesity, significantly polygenic disorders, cardiovascular disease, and heart conditions affect social and monetary systems. Over the past decade, research in drug discovery and development has opened new paths for alternative and conventional medicine. With a deeper comprehension of its underlying mechanisms, obesity is now recognized more as a chronic condition rather than merely a result of lifestyle choices. Nonetheless, addressing it solely through lifestyle changes is challenging due to the intricate nature of energy regulation dysfunction. The Federal Drug Administration (FDA) has approved six medications for the management of overweight and obesity. Seaweed are plants and algae that grow in oceans, rivers, and lakes. Studies have shown that seaweed has therapeutic potential in the management of body weight and obesity. Seaweed compounds such as carotenoids, xanthophyll, astaxanthin, fucoidans, and fucoxanthin have been demonstrated as potential bioactive components in the treatment of obesity. The abundance of natural seaweed bioactive compounds has been explored for their therapeutic potential for treating obesity worldwide. Keeping this view, this review covered the latest developments in the discovery of varied anti-obese seaweed and its bioactive components for the management of obesity.

## 1. Introduction

A World Health Organization (WHO) report indicates that more than 600 million people are now affected by obesity globally [1]. The report on obesity in Korea shows that the male obesity rate decreases with age (especially over 70 years), while the female obesity rate increases rapidly with age [2]. It has been observed that obesity is the result of the imbalance between intake and expenditure of energy in the body, and it is a state of chronic, proinflammatory process that initiates from the surplus adipose tissue [3]. Obesity is also defined as excess accumulation of intra-abdominal body fat, which may be divided into two categories: subcutaneous adipose tissue (SAT) and visceral adipose tissue (VAT). VAT may be a stronger predictor of obesity connected with inflammation and metabolic disorders [4]. Adipocytes store excess fats and triacylglycerols, which serve as energy reserves. When the body needs energy, these reserves are broken down, releasing free fatty acids that can be used for fuel. The storage of triacylglycerols also forms adipokines, a key cytokine of adipose tissue involved in inflammation, immunity, and energy metabolism. The adipokines affect glucose metabolism in the liver, muscles, and fat tissues [5]. Additionally, obesity induces chronic low-grade inflammation, which can negatively impact the quality of life and cast an additional burden on healthcare expenses.

The association between obesity and colorectal cancer was evaluated in the last decade but, in 2022, obesity’s association with breast cancer and lung cancer has also been observed [6,7]. Allopathic medicine, which is being used for the reduction in obesity, also has side effects and produces metabolic disorders. Therefore, it is necessary to find an alternative medicine along with conventional therapy for better treatment of obesity. In the latest study in 2024, the effect of various kinds of exercise and yoga on obesity was explained [1]. Now, in the path of alternative medicine, seaweeds might play an important role and provide a new direction as supplementary materials in treating obesity. In the current review, the significance of seaweeds in treating obesity is discussed with the latest conventional therapy.

Seaweeds are autotrophic organisms found in coastal areas and are an excellent source of protein, vitamins, essential fat, fibers, and omega-3 and omega-6 polyunsaturated fatty acids [8]. There are several types of seaweeds; green and red seaweeds contain higher protein than brown–green seaweeds, while polyphenols are higher in brown seaweeds as compared to red seaweeds [9]. Seaweeds can be classified taxonomically into three major groups: Chlorophyta (green algae), Phaeophyceae (brown algae), and Rhodophyta (red algae). Seaweed is the traditional food of many countries, including China, Japan, Korea, Mexico, and other countries [9]. Fiber is a copious important element for healthy food and it can be classified into soluble and insoluble fiber. Soluble fibers are responsible for gel formation in contact with water, in the intestine, while insoluble fiber is not capable of making gel. Insoluble fiber is capable of retaining water, acts as a laxative in the digestive tract, and accelerates fecal intestinal movement [10]. Soluble fiber decreases blood cholesterol and glucose concentration and promotes the growth of intestinal microbiota [11]. Seaweeds contain primary and secondary metabolites; primary metabolites like carbohydrates and fiber help reduce cardiovascular disease and obesity, while secondary metabolites fight a stressful situation. Secondary metabolites such as polyphenols and fucoxanthin have been seen to suppress hunger and reduce obesity.

The major diseases or disorders related to obesity are metabolic syndrome, high blood pressure, dyslipidemia, myocardial infarction, stroke, and specific cancers. Obesity patients are simply frustrated throughout their life and feel anxiety, which reflects on their faces. In the present review, the impact of various anti-obesity seaweed and bioactive ingredients food on obesity has been studied.

## 2. Seaweeds as Anti-Obesity Weapons

Seaweed is a good source of fiber and highly unsaturated fatty acids (HUFA) such as alpha-linolenic acid (18:3n-3) and LA (linoleic acid, 18:2n-6). These fatty acids are called essential fatty acids as they are necessary for our health development but are not synthesized in our body [12,13]. In our body, these essential fatty acids change into long-chain fatty acids. These long-chain fatty acids, such as arachidonic acid, are important for brain development and reduce obesity. The latest research in 2022 shows the reduced level of inflammatory markers and cytokines in obese mice [14]. Sourav Roy et al. provided arachidonic acid supplementation to high-fat-diet-induced mice and observed that cytokine gene expression reduced in obese mice, although the study shows no significant changes in the level of triglyceride. In *Palmaria palmata* (red algae), essential fatty acid changes *into eicosatetraenoic*, while, in *Gracilaria gracilis*, it changes into *Arachidonic acid.* Additionally, seaweeds also contain various bioactive compounds such as pigments and minerals, which may reduce pathological conditions of diseases [15]. Epidemiologic studies showed that intake of seaweeds reduces the causes of the onset of lipidemia, cancer, and type 2 diabetes [16].

*Fucoxanthin* is one of the pigments obtained from seaweeds that reduce the absorption of triglyceride in GIT and reduce obesity [17]. *Alginates*, another cluster of algal compounds, possess inhibitory activity against exocrine gland enzymes. The repressing activity of alginates might vary in different kinds of algae and also depends on the composition of various alginates [18]. El Khoury and colleagues investigated how adding sodium alginate to chocolate milk affected blood glucose and insulin levels. They found that 2.5% alginate-added milk reduced the 6% glucose peak and 46% insulin peak at 30 min compared to chocolate milk [19].

### 2.1. Green Algae Carotenoid Siphonoxanthin and Neoxanthin

Siphonoxanthin has been identified as a bioactive compound in green algae such as *Caulerpa lentillifera*. The anti-obesity effect of Siphonoxanthin in 3T3 cells has been evaluated and it was found that it prevents lipid accumulation and adipogenesis. Siphonoxanthin, a compound found in green algae, can stimulate energy expenditure and decrease fat storage in white adipose tissue (WAT) by altering gene expression, which can inhibit lipid synthesis in WAT and potentially prevent obesity. Apart from the anti-obesity effect, it also contains anti-cancerous activity, as it enhances the antiangiogenic impact on cancer cells [20]. It also inhibits kinase B phosphorylation in 3T3-L1 cells and reduces the gene expression of CCAAT-enhancer-binding protein α (C/EBPα), peroxisome proliferator-activated receptor γ (PPARγ), fatty-acid-binding protein 4 (Fabp4), and stearoyl-CoA desaturase 1 (SCD1) [21].

Another carotenoid present in green algae and plants is Neoxanthin. It is important xanthophylls are found in algae along with terrestrial plants. Neoxanthin is found in two isomeric forms; one is 9′-cis-neoxanthin and another is trans-neoxanthin. 9′-cis-neoxanthin is found in green leaves abundantly where photosynthesis occurs, while trans-neoxanthin is found in fruits and flower petals [22]. An in vitro study on 3T3 cells reveals that Neoxanthin inhibits lipid accumulation like Siphonoxanthin and decreases gene expression of C/EBPα and PPARγ [23].

### 2.2. Brown Seaweed Phlorotannin

*Phlorotannin* is a polyphenol that inhibits the lipid digestive enzyme, pancreatic lipase; as a result, lipid absorption is hampered and lipids are excreted as undigested food from the body. *Phlorotannin* not only reduces obesity but also contains anticancer and antiviral activity [24].

Brown seaweeds are a good source of *Phlorotannin*, a natural bioactive compound. The lipase enzyme is responsible for lipid digestion secreted in pancreatic juice, which digests lipids into fatty acids and glycerol and helps for absorption of lipids in the duodenum and jejunum (small intestine) [25]. Seaweeds can be considered potential therapeutic agents due to their rich source of bioactive compounds. Algae bioactive compounds are known for the management of digestion and have the potential capacity for obesity treatments [26].

### 2.3. Brown Seaweed Fucoxanthin

Fucoxanthin, a marine carotenoid predominantly found in brown seaweeds and diatoms, constitutes the majority of naturally occurring carotenoids. It is particularly abundant in edible seaweeds like *Undaria pinnatifida*, *Laminaria digitata*, and *Hijikia fusiformis*, as well as marine microalgae. Fucoxanthin’s potent inhibitory properties make it a promising candidate for functional food applications [27]. The anti-obesity property of fucoxanthin depends upon the fact that it modulates the transcription factors/regulators that play a crucial role in adipocyte differentiation and development [28].

**Figure 1:**
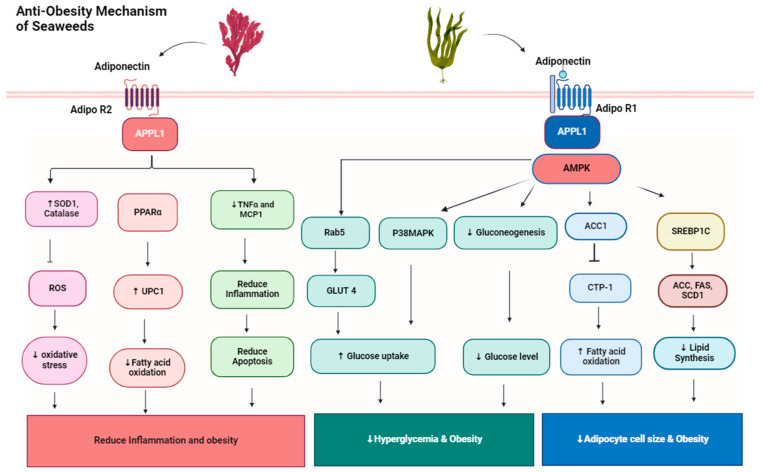
Seaweed consumption enhances the level of adiponectin hormone, which binds adiponectin receptor adipoR1 and Adipo R2 in the liver and maintains lipid and glucose homeostasis. In liver adipocyte cells, when adiponectin binds AdipoR1, it maintains homeostasis by the AMPK pathway. Adiponectin decreases hepatic lipogenesis and increases B-oxidation through the AMP protein kinase pathway. Phosphorylated-AMPK inhibits lipogenesis by (1) suppressing SREBP1c expression and (2) phosphorylating acetyl CoA carboxylase-1 (ACC-1), which inhibits carnitine palmitoyl transferase-1 (CPT-1) activity and enhances fatty acid transport into the mitochondria to undergo B-oxidation. Adiponectin also inhibits hepatic gluconeogenesis, independent of AMPK, decreasing glucose output and improving glycemia. APPL1 activates through the Adipo R1 receptor and activates Rab5, which is synthesized in endosomes and transported to the adipocyte cell membrane, which allows GLUT 4 transport in adipocyte cells and increases glucose consumption; as a result, glucose levels in the blood decrease. When adiponectin binds AdipoR2, it upregulates the level of UPC2 in the mitochondria of adipocytes through peroxisome proliferator-activated receptors (PPARα) pathway cells in the liver and decreases fatty acid oxidation and reduces obesity. It also increases superoxide dismutase and catalase enzymes, which inhibit reactive oxygen species (ROS) and reduce stress which causes obesity.

In adipocytes, various nuclear protein receptors are expressed during early development, which regulate adipogenesis. Sterol regulatory-element-binding protein1c (SREBP-1c) is one of the nuclear protein receptors that regulate fatty acid and cholesterol synthesis [29]. In another pathway, fucoxanthin upregulates the uncoupling protein1 in brown adipose tissue. The uncoupling protein1 increases fat oxidation in mitochondria in adipose tissue and enhances thermogenesis. The mechanism of the effect of seaweeds on obesity is illustrated in Figure 1.

An in vitro study by Maeda et al. analyzed the anti-obesity effect of fucoxanthin in 3T3-L1 cells and found that fucoxanthin prevented the deposition of lipids inside the 3T3-L1 and stopped the conversion of fucoxanthin to fucoxanthinol. The 3T3-L1 cell line was created in 1962 by modifying the original 3T3 Swiss albino cell line. It is used to investigate diseases and abnormalities associated with adipose tissue. Fucoxanthin also reduces lipid differentiation and development in 3T3-L1 cells [30]. The effect of fucoxanthin on body weight is still unclear. Some studies have shown that fucoxanthin can help mice and rats gain weight when they are fed a high-fat diet, while other studies have found no significant difference. The study was investigated to see the potential benefits of fucoxanthin-rich wakame lipids (68% glycoloipid + 10% fucoxanthin) in combating obesity in mice. Mice were provided a high-fat diet and compared with a normal-fat diet for 10 weeks. The study found that fucoxanthin reduces body weight along with the weight of white adipose tissue [31]. The study is also supported by another study carried out in 2015 by Maria et al. [32].

### 2.4. Brown Seaweed Alginates

Alginate or alginic acid is a naturally edible polysaccharide known for its anti-obesity effects. Alginate’s derivatives of algin copolymerize with β-1,4-d-mannuronic acid and α-1,4-l-guluronic acid to form the structural part of brown seaweeds. The role of sodium alginate, a dietary fiber derived from brown seaweed, in weight loss has been observed [33]. However, the exact mechanism of how alginate reduces body weight is still not clear. In one study, when alginate was provided to overweight people (15 g/day) for 14 weeks, it reduced body weight in comparison to maltodextrin [34]. Alginate forms gel in the stomach due to its viscous nature, giving the feeling of satiety and reducing appetite [35]. Eating alginate bread was associated with lower levels of circulating triacylglycerols compared to a control diet. Alginate may help reduce lipid absorption by inhibiting pancreatic lipase, an enzyme that is involved in fat breakdown [36]. Alginate may change the gene expression related to lipid metabolism and show the weight loss effect. The latest research indicates the role of microbiota in maintaining health and reducing obesity and related disorders. It is believed that alginate can help prevent weight gain by improving gut microbiota [37].

### 2.5. Brown Seaweed Fucoidans

It is an extremely sulfated polysaccharide found in the cell wall and extracellular space between the cells in brown seaweeds. The major sugar present in fucoidans is fucose, which is found in pyranose form. Like the other polysaccharides, it is not linked with α-[1,4] or α-[1,6] glycosidic bonds, although it is α-(1–3)-linked. Fucoidans have anti-inflammatory and wound-healing properties [38]. Few studies validate that fucoidans may inhibit obesity. The antiadipogenic activity of fucoidans has been seen in *Undaria pinnatifida* brown seaweed. Moreover, fucoidans prevent the accumulation of lipids and reactive oxygen species (ROS) in adipocytes. An in vitro study revealed that fucoidans were additionally found to inhibit lipid deposition in cell line 3T3-L1 cells [39,40]. Fucoidans downregulate the gene expression related to adipogenesis in the 3T3-L1 cell line. The study indicates that fucoidans may reduce obesity by either preventing the deposition of fat or promoting lipolysis.

### 2.6. Red Seaweed

*Campylaephora hypnaeoides* J. Agardh (*C. hypnaeoides*) is a very famous red seaweed in Japan. It is also used as a local dish known as Egonori in Japan and contains high polysaccharides. It is found near the coast of the Japan Sea. This study reveals that it prevents postprandial hyperglycemia. In the mice study, it was observed that, when the red seaweed was given to HF-diet-induced obese mice for 13 weeks, the HF diet supplemented with 2% *C. hypnaeoides* and 6% *C. hypnaeoides* reduced body weight, decreased insulin resistance, decreased adiponectin, and increased malondialdehyde (MDA), tumor necrosis factor α (TNF-α), and monocyte chemoattractant protein-1 (MCP-1) [41]. It also decreased glutathione and superoxide dismutase, which increase oxidative stress. In this way, it suppressed inflammatory response and increased antioxidant response. This study reveals that red seaweeds have anti-obesity therapeutic potential; however, more human trials are required to ascertain the facts.

### 2.7. Astaxanthin (ASX)

**Figure 2:**
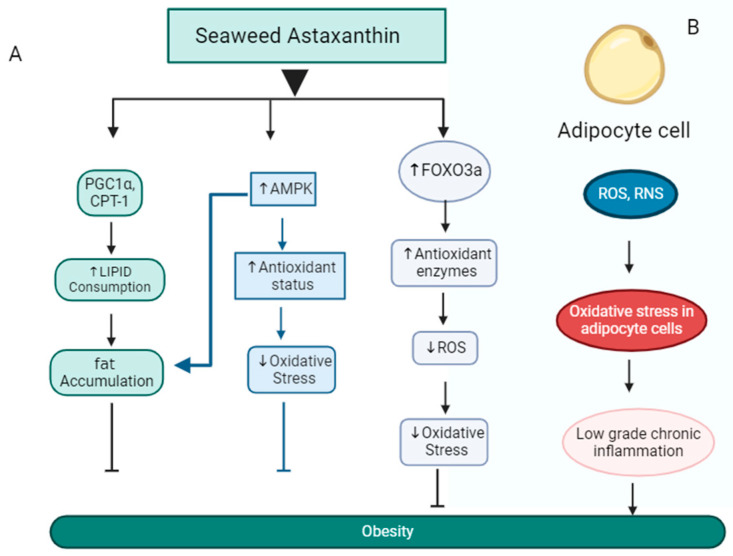
The proposed mechanism of seaweed astaxanthin’s effects on obesity. (**A**) Astaxanthin activates the AMP-activated protein kinase (AMPK) pathway and FOXO3A, leading to increased antioxidant enzyme activity in adipose tissue and reduced oxidative stress. This ultimately contributes to weight loss. Additionally, astaxanthin increases the expression of peroxisome proliferator-activated receptor gamma coactivator 1-alpha (PGC-1α) and carnitine palmitoyl transferase 1 (CPT-1); CPT-1 is a gatekeeper enzyme present in the mitochondrial membrane of adipose tissue, which allows fatty acid transport inside the mitochondria for B oxidation and prevents the accumulation of fat and further obesity, while peroxisome proliferator-activated receptor γ (PPARγ) coactivator-1 α (PGC-1α) is a key transcriptional cofactor that alters gene expression of various kinds of genes necessary for mitochondrial biogenesis in brown adipose tissue (BAT). (**B**) The production of reactive oxygen species (ROS) and reactive nitrogen species (RNS) in adipose tissue, leading to obesity. The increased levels of ROS and RNS result in oxidative stress within adipocyte cells, which contributes to low-grade inflammation and ultimately causes obesity.

It is xanthophyll carotenoids containing red pigment that have antioxidant properties [42]. It is the most potent antioxidant, exhibiting 100 times more oxygen-scavenging ability than vitamin E. It inhibits cholesterol synthesis and reduces the size of adipocytes and body weight [43]. The study demonstrates that, when astaxanthin extracted from green microalga *Haematococcus pluvialis* is taken as a supplement, it reduces body weight and prevents lipid storage in the liver, which enhances liver functions [44]. Gut microbiota analysis revealed that astaxanthin alleviated high-fat-diet-induced gut dysbiosis by restoring the Firmicutes-to-Bacteroides ratio. Additionally, astaxanthin reduces the number of obesity-associated pathogenic bacteria and induces the growth of beneficial microbiota associated with carbohydrate and lipid metabolism. Studies conducted on *Caenorhabditis elegans* have shown that astaxanthin, when combined with other compounds like anthocyanin (in nanoparticle form), can reduce lipid levels and exhibit antioxidant properties, suggesting potential benefits of astaxanthin in addressing obesity. In high-sugar-diet-induced high-fat *Caenorhabditis elegans*, these nanoparticle supplementations reduce lipid accumulation and increase life span by improving antioxidants [45]. The antioxidant property of astaxanthin is higher than other carotenoids due to the presence of two additional oxygenated groups. Astaxanthin, a potent antioxidant, neutralizes reactive oxygen species (ROS) and reactive nitrogen species (RNS), reduces the low-grade inflammation in adipose tissue, and reduces obesity [46]. The possible mechanism of astaxanthin is illustrated in Figure 2. Humans acquire astaxanthin primarily through seafood consumption or dietary supplements. Healthy adults can safely consume up to 6 milligrams of astaxanthin daily. Despite astaxanthin having several beneficial effects, few studies have produced inconsistent results regarding the actual health benefits attributed to astaxanthin.

## 3. Seaweed Edible Product

**Figure 3:**
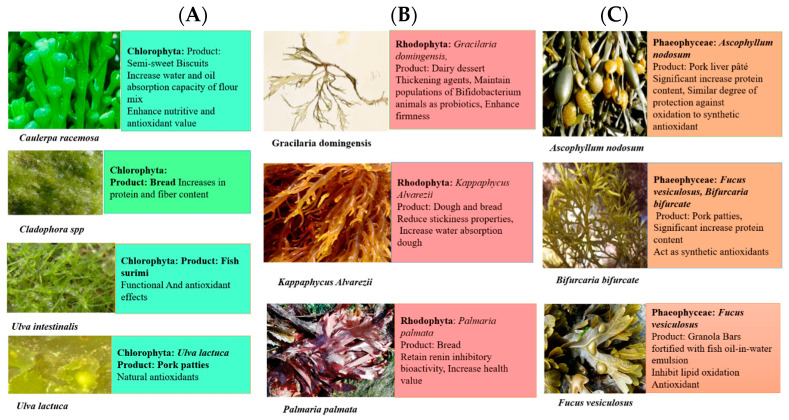
(**A**) The seaweeds of Chlorophyta and their products available in the market showing antioxidant capacity and anti-obesity characteristics. The seaweed *Ulva* spp. was used in making semi-sweet biscuits having antioxidant capacity. *Ulva intestinalis* was used for making fish surimi having antioxidant effects. *Ulva lactuca* and *Ulva rigida* were used for making pork patties having natural antioxidants and *Cladophora* spp. and *Ulva* spp. were used in bread making. (**B**) The seaweeds of Rhodophyta and their products available in the market showing antioxidant capacity and anti-obesity characteristics. The seaweed *Gracilaria domingensis* and *Crassiphycus birdiae* were used in dairy products as probiotics and thickening agents. *Kappaphycus alvarezii* was used for making dough and bread, to increase water absorption in the small intestine. *Palmaria palmata* were used for making bread to enhance nutritive value. (**C**) The seaweeds of Phaeophyceae and their products available in the market. The seaweed *Ascophyllum nodosum* was used in making pork liver pâté having antioxidant capacity. *Fucus vesiculosus* and *Bifurcaria bifurcate* were used for making pork patties having antioxidant activity and enhancing protein content in patties. *Fucus vesiculosus* was used for making granola bars fortified with fish oil-in-water emulsion that inhibit lipid oxidation and act as antioxidants.

Milk products like milk dessert, yoghurt, and smoked cheese are currently being used to mix brown algae Laminaria. Smoked cheese is a cheese that has been preserved using smoke-curing. There are two main methods of smoke-curing: cold-smoking, which involves exposing the food to smoke at temperatures between 20° and 30 °C, and hot-smoking, which partially or fully cooks the food while treating it with smoke at temperatures ranging from 40° to 90 °C. Alginate oligosaccharides of Laminaria hyperborean are being used as supplements in yogurt [47]. The nutritional value of milk products can be enhanced by adding seaweed. The brown algae *Ascophyllum nodosum* and *Fucus vesiculosus* have been used as antioxidants to enhance the life of milk and quality [48]. *Saccharina latissimi* use as iodine supplementation has been observed in cheese. Cardiovascular diseases such as hypertension and others can be prevented by adding seaweeds to food content as their application in food reduces sodium content [49]. Due to acting as an antioxidant and having Angiotensin-I-converting enzyme inhibitory activity, *Palmaria palmate* and *Saccharina longicruris* are also used as flakes. *P. palmata* is a very good source of protein and carbohydrate contents, while *S. longicruris* has an excellent source of fiber and mineral contents, which makes it a very promising food [50]. *Undaria pinnatifida and Laminaria ochroleuca* are being used to develop gluten-free products [51]. The seaweeds and their edible products with their beneficial effects are illustrated in Figure 3.

### 3.1. Japanese Famous Edible Seaweed

**Figure 4:**
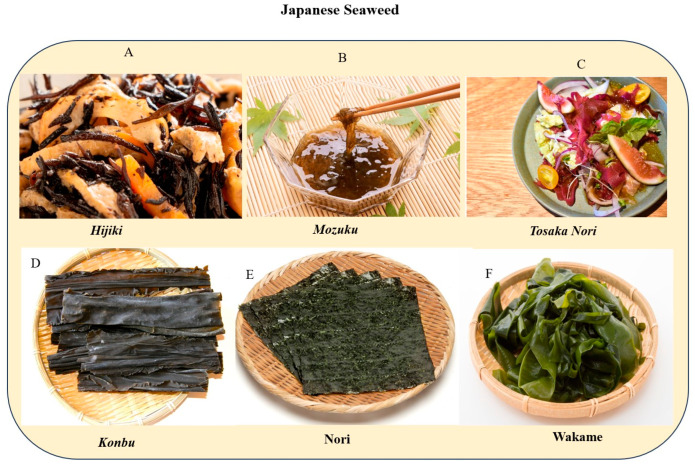
The most common Japanese seaweeds. (**A**) Hijiki (rehydrated, dried) is often used in a stir-fried dish called nimono, typically with carrots, aburaage (deep-fried tofu), soybeans, and shiitake mushrooms. (**B**) Mozuku offers a chewy texture and contains fucoidan, a beneficial dietary fiber. Vinegared mozuku is packed with nutrients and aids in calcium absorption. (**C**) Tosaka nori can be used as a colorful and nutritious garnish for sashimi, adding vibrancy and beauty to salads. (**D**) *Kombu* is a good source of glutamic acid, an acidic amino acid responsible for the taste of umami. Kombu is sold in dried form called *dashi konbu* or pickled form in vinegar (*su konbu*) or as a dried shred (*oboro konbu*, *tororo konbu*, or *shiraga konbu*). (**E**) The main types of nori sold in Japan are *susabinori* and *asakusanori*. It comes in three forms and is known as *namanori* (freshly taken from the sea), while the dried version is *kannori*; when grilled, it is known as *yakinori*. (**F**) Wakame leaves are green and have a sweet flavor, containing fucoxanthin, which may help burn fatty tissue. Wakame is also a good source of Eicosapentaenoic acid, an omega-3 fatty acid.

In Japan, seaweeds like Mozuku, Hijiki, Wakame, and Nori are highly prized for their nutritional value and health benefits. These seaweeds are popular choices due to their potential to lower blood pressure, reduce the risk of cardiovascular diseases, and aid in weight management and metabolic health. The Japanese seaweeds are illustrated in Figure 4.

Mozuku

Mozuku, a type of seaweed, is primarily farmed in Okinawa, where it is known for its chewy texture and slightly slimy consistency. Residents of Okinawa Prefecture exhibited significantly higher consumption of Mozuku compared to those residing outside the prefecture [52]. The average Japanese adult consumes approximately 14.3 g of seaweed per day [53].

In contrast, Itomozuku, found in the Hokuriku region, is significantly slimmer with finer strands. Mozuku contains fucoidan, a soluble dietary fiber that can be easily absorbed when added to miso or other soups. Consuming Mozuku with vinegar and soy sauce can enhance calcium absorption due to the citric acid in the vinegar. This nutritious seaweed offers a refreshing and smooth texture, making it a popular side dish.

Hijiki

Hijiki, a type of seaweed, is typically sold in two forms: soft, bud-like mehijiki and chewy, long-stemmed nagahjiki. While both fresh and dried hijiki are available, the majority of hijiki sold in Japan is imported. Dried hijiki is often rehydrated and cooked as nimono, a simmered dish that often includes carrots, aburaage (deep-fried tofu), soybeans, and shiitake mushrooms. Hijiki can be incorporated into various Japanese dishes, such as rice dishes, salads, tamagoyaki (rolled omelet), tempura, shiraae (mashed tofu salad), sunomono, and miso soup. Renowned for its high calcium content, hijiki is a valuable food for those seeking to address calcium deficiencies. Hijiki algae extract showed the weakest antimicrobial activity [54]. Hijiki seaweed contained the highest amount of calcium, with a concentration of 8853.82 milligrams per kilogram [55]. The study also indicated that cobalt is present in hijiki and kombu. Hijiki fusiformis demonstrated the strongest antioxidant activity, reaching 65% [56].

Wakame

Wakame, a type of seaweed, is a popular ingredient in Japanese cuisine. It is prized for its nutritional value, low-calorie content, and ability to aid digestion. Wakame is often found in dried form but it can also be purchased fresh, cooked, salted, or charcoal-dried. While most wakame available in Japan is farmed, the town of Fukaura in Aomori specializes in natural wakame harvested from the ocean. They use a traditional drying method called yaki-boshi-hai, which involves baking and drying the seaweed in ash. This process helps preserve the seaweed’s bright color, flavor, and aroma.

Wakame was found to have a significant acute effect on postprandial blood glucose and insulin levels. When consumed with rice, wakame reduced both blood glucose and insulin levels within 30 min compared to consuming rice alone [57]. Wakame is rich in minerals like calcium, iron, phosphate, fiber, and vitamin K. These nutrients make it a beneficial food for bone health and digestion. To maximize mineral absorption, it is recommended to season wakame with vinegar. Due to its low-calorie content, wakame is a popular choice for those on diets. Wakame is helpful in the prevention of metabolic syndrome [58].

Nori

Nori, a popular seaweed in Japan, is primarily sold in two main types: susabinori and asakusanori. It is available in three forms: fresh (namanori), dried (kannori), and grilled (yakinori). Most nori sold in stores is grilled. To produce dried and grilled nori, fresh sheets of seaweed are spread out like washi paper and allowed to dry. Nori has numerous culinary uses, serving as a wrap for sushi, onigiri, and isobemochi (grilled rice cakes). It is also an ingredient in furikake seasoning and a topping for zaru soba (chilled noodles) and ramen. Flavored nori, made from grilled nori coated with sauce, is a popular accompaniment to rice.

Nori was used to prepare sauce by fermentation procedure and compared with soy and fish sauces, indicating a rich source of potassium and nitrogen [59].

Approximately 40% of nori demand is for home consumption and gifting, while 60% is for processing. The majority of processed nori is used in onigiri sold at convenience stores. Fresh nori finds its way into dishes like tsukdani (a preserved food) and miso soup and can be added to pasta. Often referred to as a “vegetable of the sea”, nori is rich in vitamin C, protein, and dietary fiber. Consuming nori (dried laver) was associated with lower diastolic blood pressure levels in boys, suggesting that seaweed intake may help prevent hypertension in childhood [60]. Nori, a type of red seaweed commonly cultivated in Asia, has been found to enhance the immune response in mouse macrophages. Extracts from nori, scientifically known as *Porphyra tenera*, were shown to regulate the NF-κB IκB kinase (IKK) signaling pathway, a key pathway involved in immune function [61].

### 3.2. Preclinical and Clinical Evaluation on the Efficacy and Safety of Seaweed for Obesity Treatment

The U.S. Food and Drug Administration (FDA) and the European Medicines Agency (EMA) follow a similar four-phase drug development process. Each phase involves multiple stages, ensuring the safety and efficacy of new medications as follows: (1) Discovery and Development: identification of potential drug candidates through research and screening; characterization of the molecular targets and mechanisms of action. (2) Preclinical Research: laboratory testing (in vitro) to assess the drug’s properties and interactions with biological molecules; animal studies (in vivo) to evaluate safety, efficacy, and pharmacokinetics. (3) Clinical Trials: Phase I: small-scale studies in healthy volunteers to assess safety and tolerability. Phase II: larger studies in patients with the target disease to evaluate efficacy and identify optimal dosage. Phase III: large-scale clinical trials to confirm efficacy, safety, and optimal dosage in a diverse patient population. (4) Regulatory Review and Approval: submission of comprehensive data to regulatory agencies for review; evaluation of safety, efficacy, and quality standards; approval for commercialization if the drug meets regulatory requirements. (5) Post-Market Surveillance: ongoing monitoring of the drug’s safety and efficacy after it enters the market; identification and reporting of adverse events; potential for updates to the drug’s label or restrictions on use.

To establish seaweed compounds as drugs, rigorous preclinical and clinical research is indispensable. These studies aim to characterize the compound’s chemical structure; assess its biochemical properties, including stability and reactivity; and evaluate its pharmacological activities, such as potency, toxicity, and selectivity. If these studies yield promising results, the compound can proceed to pharmacodynamic and pharmacokinetic studies. These assess how the drug interacts with the body and how it is absorbed, distributed, metabolized, and excreted.

Numerous preclinical and clinical studies have explored the potential of seaweed compounds for treating obesity. Key findings include limited clinical trials: many seaweed compounds remain in preclinical stages, with relatively few progressing to clinical trials; varied methodology: the wide range of methodologies used in preclinical studies has hindered the advancement of some compounds; and regulatory challenges: several seaweed compounds have not been approved by drug regulatory agencies, limiting their clinical development. While seaweed compounds offer potential therapeutic benefits, significant research and regulatory hurdles remain to be overcome before they can be established as effective drugs. Continued efforts are needed to advance the development of seaweed-derived therapeutics for diabetes and other diseases.

## 4. Conventional Treatment of Obesity

The treatment of obesity by medicine has attracted a lot of attention from clinicians and patients, though the quantity of additional weight loss owing to it is modest (<5 kg at 1 year) [62]. This amount of weight loss has been shown to enhance glycemic management, dyslipidemia, hypoglycemic agent sensitivity, and high blood pressure in overweight patients. Several factors enhance obesity. According to the latest study published in Lancet 2023, Metreleptin and Setmelanotide are currently used for rare obesity syndromes, while five other medications (orlistat, phentermine/topiramate, naltrexone/bupropion, liraglutide, and semaglutide) are approved for non-syndromic obesity by the FDA [63].

Metreleptin

Metreleptin (AstraZeneca plc, Cambridge, UK) is a synthetic form of leptin approved by the FDA in 2014 for use as a replacement therapy in patients with congenital or acquired lipodystrophy who have a deficiency in leptin production. Metreleptin is administered as a daily subcutaneous injection. The starting dose ranges from 0.06 mg/kg/day to 0.13 mg/kg/day, depending on the severity of obesity. Metreleptin shows its potential effect around 6 months later; the study published in the Lancet indicated that it reduces cholesterol, triglyceride, and urinary glucose at six months [63]. It also decreases lean body mass and body weight at six months.

Setmelanotide

Setmelanotide,(Rhythm Pharmaceuticals, Inc., Boston, MA, USA) a melanocortin-4 (MC4) receptor agonist, was approved by the FDA in 2020 for chronic weight management. It is administered as a subcutaneous injection and is indicated for patients aged 6 and older with obesity caused by genetic deficiencies in proopiomelanocortin or the leptin receptor (LEPR). The failure of activation of the MC4 pathway enhances hunger and causes childhood-onset obesity. Furthermore, setmelanotide enhances MC4 receptor signaling, reduces hunger, and enhances body weight loss by increasing energy expenditure.

Phentermine/Topiramate

Phentermine/Topiramate (Qysmia^,^ Vivus pharmaceutical Ltd., Campbell, CA, USA) was approved by the FDA in 2012 for the chronic management of obesity. However, due to severe neuropsychological side effects, it was not approved by the European Medical Agency (EMA). The memory-related issue was also noticed as a side effect in obese patients [64].

Naltrexone/Bupropion

Naltrexone/Bupropion, (Orexigen Therapeutics, Inc., La Jolla, CA, USA) (2014/15 FDA approved) acts as a dopamine (DA) and NE reuptake inhibitor along with opioid receptor antagonist, used in bulimia or anorexia nervosa and alcohol dependence. Obesity is associated with the alteration of proopiomelanocortin (POMC) neurons and the reward system of the hypothalamus. NB activates (POMC) neurons in the hypothalamus and increases the production of POMC peptide hormone, which ultimately reduces food intake and weight loss [65].

Semaglutide

Semaglutide (Wegovy, Novo Nordisk, Patel Nagar, New Delhi, India), a GLP-1 analog approved by the FDA in 2021, is used to treat obesity. However, it is not recommended for individuals with a personal or family history of medullary thyroid carcinoma, those with multiple endocrine neoplasia syndrome type 2, or pregnant women.

Tirzepatide

Tirzepatide,(Eli Lilly and Company, Indianapolis, Indiana) received FDA approval, 8 November 2024) for obesity treatment.The novel medicine used for the treatment of obesity, is not used in type I diabetes and severe gastrointestinal disease. Although, it is recommended for type II diabetes [63]. Tirzepatide stimulates insulin secretion from the pancreas, helping to lower blood sugar levels. Additionally, it increases adiponectin levels. Tirzepatide is a 39 amino acid peptide that reduces hunger and decreases hyperglycemia. It has a half-life of 5 days and reaches a maximum level in serum 2 in 3 days [66]. Based on their mechanism of action, the drug treatment of obesity is classified into three categories: (a) medicine that interferes with fat absorption, (b) medicine that increases energy expenditure and thermogenesis, and (c) medicine that cuts back food intake.

### 4.1. Medicine That Interferes with Fat Absorption

Sibutramine and orlistat are copious important drugs that are included in this category.

#### 4.1.1. Orlistat

Gastric and pancreatic lipases are key enzymes of fat digestion in humans and Orlistat is a selective inhibitor of gastric and pancreatic lipases. It acts on serine residue, which is part of the active site present in these enzymes [67,68]. It prevents the hydrolysis of triglyceride into monomer-unit monosaccharide, which is absorbed in the intestine. Orlistat is a derivative of lipstatin isolated from actinomycete toxytricini [69]. It is an artificial drug designed to act on a target site and block the absorption of dietary fat [70]. Orlistat may be an artificially modified by-product of lipstatin that can be used in the regulation of body weight control [71]. Orlistat is a medication that works by blocking the absorption of dietary fat. Common side effects include flatulence, bloating, abdominal pain, and indigestion, which are often caused by undigested fat passing through the digestive system [62]. Patients taking orlistat should be monitored for nutrient deficiencies, as the medication can reduce fat absorption, leading to a potential shortage of fat-soluble vitamins (A, D, E, and K). A study on obesity found that orlistat treatment was associated with significant reductions in waist circumference, total cholesterol, LDL cholesterol, and blood pressure and improvements in glucose levels and insulin sensitivity [72].

#### 4.1.2. Lipstatin

Lipstatin, a compound initially isolated from the actinobacterium *Streptomyces toxytricini*, contains a β-lactone ring responsible for its irreversible enzyme inhibition [73]. The potent inhibitory action of orlistat has raised concerns about its potential to interfere with the absorption of certain vitamins. Extensive research is underway to identify extracts from diverse natural sources, including plants, fungi, algae, and bacteria, that exhibit inhibitory activity against pancreatic lipase [74].

#### 4.1.3. Sibutramine

Sibutramine is a dual monoamine reuptake inhibitor, affecting both norepinephrine and serotonin. It is believed to promote modest weight loss by reducing appetite and increasing energy expenditure. Compared with alternative agents’ adverse effects are restricted; however, flatulence, bloating, abdominal pain, and indigestion [75] side effects from anti-obesity medicine are a significant concern for their therapeutic usage. Therefore, sibutramine is the first drug opposing obesity that has been withdrawn from the market because of the aspect of vessel events and strokes [76].

### 4.2. Medicine That Increases Energy Expenditure and Thermogenesis

Ephedrine and caffeine can be included in this category. One future placebo-controlled test with caffeine and ephedrine or their combination shows that the mixture of caffeine and ephedrine greatly affected weight loss compared to individual use. These substances are contained in some health supplements. β3-AR agonist in mice increases UPC1 protein expression in brown adipose tissue (BAT), induces thermogenesis, and ultimately decreases body weight [77]. In humans, Mirabegron increases ATGL and UCP1 protein in the mitochondria of BAT and also increases insulin sensitivity [78]. β3-adrenergic receptor agonists recently emerged as novel pharmacological therapeutics to counteract obesity and other metabolic diseases [79].

### 4.3. Medicine Suppressing Hunger and Satiety

Anti-obesity medications include noradrenergic, serotonergic, and serotonergic–adrenergic drugs. Some of these medications target the gamma-aminobutyric acid (GABA) or cannabinoid receptors, while others are peptides that reduce appetite or promote feelings of fullness [80]. Many weight-management medications target neurotransmitters in the central nervous system to reduce appetite. Noradrenergic drugs, which either release or block the reuptake of the neurotransmitter norepinephrine, can affect food intake. These medications work by interacting with beta-adrenergic receptors in the peripheral hypothalamus, ultimately reducing appetite. Examples of noradrenergic medications include phentermine and diethylpropion [81].

#### 4.3.1. Phentermine and Diethylpropion

Diethylpropion, also known as generic Tenuate, is a deep-rooted medication that has been used to treat obesity for almost 60 years. The Federal Drug Administration (FDA) approved it as an anti-obesity medicine in 1959 as it decreases the sensation of hunger. Many appetite-suppressant medications target noradrenergic and potentially dopaminergic receptors in the brain to promote feelings of fullness. However, weight loss by amphetamine is sustained till 36 months. Comparative studies have shown that phentermine may lead to greater weight loss than diethylpropion over 12 weeks [82,83]. Both medications can cause side effects such as headache, insomnia, irritability, palpitations, and nervousness. However, there is a need for more research to fully understand the effectiveness and safety of phentermine and diethylpropion.

#### 4.3.2. Dexfenfluramine and Fenfluramine

These are serotonin-enhancing medications that work by increasing serotonin levels in the brain. This can lead to a feeling of fullness and reduced appetite, particularly for fatty foods. However, these drugs have been associated with serious cardiovascular side effects, including pulmonary hypertension and heart valve damage [84]. Fluoxetine, a medication commonly used to treat depression, is a selective serotonin reuptake inhibitor (SSRI). It has a wide range of clinical applications for treating depression and has not been linked to pulmonary hypertension or heart valve damage.

#### 4.3.3. Glucagon-like Peptide-1 (GLP-1)

This is an incretin hormone released in response to glucose in the gut. It slows down stomach emptying and stimulates the release of insulin [85]. Though, it causes nausea, reduces appetite, and facilitates weight loss. Incretins are hormones secreted from the epithelium of the small intestine in response to glucose or fat molecules belonging to the glucagon superfamily. In humans, gastric inhibitory peptide (GIP) and glucagon-like peptide-1 (GLP-1) are two major incretins. When blood sugar levels rise, these incretins, secreted from the small intestine, travel to pancreatic beta cells and stimulate the release of insulin, helping to regulate blood glucose levels [86]. Liptins Increase the Levels of Incretins by Blocking Dipeptidyl Peptidase-4 (DPP-4), an enzyme that breaks down incretins and other peptides, and exenatide, an approved Anti-Diabetic Medication, Mimics the Effects of GLP-1 and Has Been Shown to Promote Weight Loss [54,87]. Liraglutide, Another GLP-1 Analog, Has Been Found to Improve blood sugar control and lead to weight loss in patients with type 2 diabetes [88].

Natural bioactive compounds derived from plants and plant products, such as herbs, fruits, and vegetables, are increasingly explored as potential ingredients in anti-obesity products. Due to increasing health awareness among people, these natural plant-based supplements are expected to play a significant role in the development of nature-sourced weight loss solutions.

These allopathic medications alter weight by increasing energy expenditure, suppressing appetite, or inhibiting the secretion of enzymes from exocrine glands to reduce the absorption of macronutrients in the intestines [89]. Weight loss medicine could seem to be an answer to obesity. Medicine for obesity ought to be meant just for patients with BMI > 30 or BMI > 27 with comorbidity [90]. Some conventional obesity medications with mechanisms of action have been illustrated in the Table 1.

#### 4.3.4. Comparison of Seaweed and Conventional Medication on Obesity

Obesity is a major health risk, linked to metabolic syndrome, which includes type 2 diabetes, high cholesterol, high blood pressure, and heart disease. The mechanism of action of conventional drugs is based on signaling pathways related to adipogenesis. Adipogenesis involves multiple signaling pathways, including those activated by insulin and IGF-1 (PI3K/AKT and MAPK/ERK), Wnt/β-catenin, AMPK, Hedgehog (Hh), and bone morphogenic protein (BMP). PPARγ regulates gene expression involved in adipogenesis, glucose and lipid metabolism, inflammation, and other biological processes. The PPARγ2 isoform is predominantly found in adipose tissue and is crucial for fat cell development and maintenance. Activating PPARγ can increase the number of small, insulin-sensitive fat cells and boost adiponectin production, leading to improved insulin sensitivity in the liver and muscles.

Natural products can work in various ways, such as blocking nutrient absorption, reducing fat cell formation, increasing energy expenditure, suppressing appetite, and altering gut bacteria composition. Although a lot of research is required for the comparison of seaweed and conventional medication, nevertheless, the possible comparisons are illustrated in Table 2.

Apart from seaweed, a lot of other natural substances’ roles in obesity have been evaluated earlier, for example, flavanols such as quercetin’s role in obesity has also been evaluated. They promote the programmed cell death process in adipocyte cells to reduce adipogenesis [97]. Green tea also shows an anti-obesity effect indirectly due to the presence of antioxidant properties of polyphenols [98]. Caffeine, another bioactive compound found in tea leaves, stimulates the somatic nervous system and works with catechins to increase energy expenditure and fat oxidation.

Nowadays, the combination of turmeric and mulberry leaf is used by medical practitioners to treat obese patients [27]. Polyphenols have demonstrated beneficial effects in combating obesity. Dietary polyphenols may help prevent fat accumulation by influencing adipocyte metabolism [99]. Flavonoids, a class of polyphenols widely found in nature, have shown promising anti-obesity properties. Plant stanols and sterols are natural compounds that block the absorption of enteral carboxylic acid and bad cholesterol like LDL and reduce weight gain in animal tests. Vegetable oils like corn, soybean, and vegetable oil are the main sources of plant stanols (Table 2).

**Table 2:** Seaweed bioactives vs. conventional therapies: a comparative analysis. This table outlines the bioactive compounds extracted from various seaweed species, their mechanisms of action, and the corresponding conventional treatments for similar conditions.

Type of Seaweed	Bioactive Compounds	Mechanism of Action as Anti-Obesity	Similarity and Comparison with Conventional Drugs (FDA Approved)
Green seaweed (*Caulerpa lentillifera)*	Siphonoxanthin	Stimulates energy expenditure, prevents lipid accumulation and adipogenesis, decreases fat storage in white adipose tissue, altering gene expression, which can inhibit lipid synthesis in white adipose tissue (WAT) [20].	Similar to the green algae bioactive compound (Siphonoxanthin), ephedrine also enhances energy expenditure by acting adrenergic receptors [100]. Statin enhances lipolysis and decreases lipid accumulation in mature adipocytes [101].
Brown seaweed	Fucoxanthin	Reduce the absorption of triglyceride. Modulates the transcription factors/regulators of adipocyte differentiation and development [28]. Upregulates the uncoupling protein1 (UCP1) in brown adipose tissue, increases fat oxidation in mitochondria in adipose tissue, and enhances thermogenesis.	Metformin (FDA-approved drug-2022) improves the acetyl-CoA carboxylase phosphorylation and decreases in triacylglycerol levels (triglycerides). It regulates lipogenic gene expression and decreases triglycerides [102]. Fibrates act as synthetic ligands for PPARα increasing fatty acids hepatic B-oxidation.
Brown seaweed	Neoxanthin	Inhibits lipid accumulation and decreases gene expression of C/EBPα and PPARγ [22].	Statin prevents adipocyte hypertrophy by increasing the number of small adipocytes and downregulating C/EBPα, PPARγ, SREBP1, leptin, FABP4, and adiponectin [101].
Brown seaweed	Phlorotannin	Inhibit the pancreatic lipase and reduce lipid absorption [24].	Orlistat inhibits pancreatic lipase and reduces lipid absorption like phlorotannin.
Brown seaweed	Alginate	Dietary fiber reduces body weight, gives the feeling of satiety, and reduces appetite by reducing lipid absorption and inhibiting pancreatic lipase [36,37].	Similar to alginate, liraglutide also reduces appetite by downregulating AKT and PI3K pathways, upregulating AMPK, and decreasing lipogenesis in white adipose tissue [103].
Brown seaweeds	Fucoidans	May reduce obesity by either preventing the deposition of fat or promoting lipolysis [38].	Similar to fucoidans, liraglutide shows pro-lipolytic effects in human mature adipocytes [104].
Red Seaweed	Whole red seaweed	It reduces body weight, decreases insulin resistance, and decreases adiponectin [41].	Zepbound new FDA-approved medication (2023) acts on intestine (glucagon-like peptide-1 (GLP-1) and glucose-dependent insulinotropic polypeptide (GIP)) to reduce appetite and food intake.
Green microalga *(Haematococcus pluvialis)*	Astaxanthin	Inhibits cholesterol synthesis and reduces the size of adipocytes and body weight [42] Acts as a potent antioxidant that neutralizes reactive oxygen species (ROS) and reactive nitrogen species (RNS), reduces low-grade inflammation in adipose tissue, and reduces obesity [46].	Statins lower cholesterol by inhibiting the conversion of HMG-CoA to mevalonic acid [105].

## 5. Critical Analysis of Seaweed

Clinical studies indicate that seaweed compounds are generally safe and well tolerated when consumed in small or moderate quantities. However, individuals taking blood-thinning medications should be cautious, as certain seaweed compounds, such as fucoidan, possess anticoagulant properties. Moreover, certain seaweed species can contain high levels of iodine, potentially causing thyroid gland dysfunction in those with iodine sensitivity or deficiency. A European study revealed that *Palmaria palmata* consumption can elevate iodine levels in adults. Participants who consumed 5 g of *Palmaria palmata* daily for 28 days showed a notable increase in thyroid-stimulating hormone (TSH) within the normal range [106]. *Laminaria digitata* contains a significant amount of iodine, with 3.3 g providing over 4000% of the daily recommended intake. Excessive consumption of seaweed with an iodine content exceeding 45 mg/kg of dry weight can potentially impair thyroid function. Considering the widespread availability and use of *Laminaria*spp. in food products, it is essential to thoroughly analyze the iodine content of these products [107]. In Korean studies, the arsenic level in urine was found to be higher in those who were consuming seaweed. *Laminaria digitata* contains a significant amount of arsenic, with concentrations ranging from 36 to 131 milligrams per gram of dry weight [108,109]. In light of climate change, monitoring the heavy metal content of seaweed is crucial. Additionally, the presence of rare earth elements, recently discovered in Mediterranean seaweeds, should be closely observed. Excessive exposure to heavy metals can harm fetal development and lead to neurological, developmental, and endocrine disorders [110,111]. While preclinical and clinical studies show promising results, current research has limitations. Many studies are relatively small and short-term, and there is a lack of standardization in seaweed compound preparation and dosing. To confirm the efficacy and safety of these compounds, larger, longer-term studies are necessary, along with the establishment of optimal dosing and preparation methods.

Very limited studies have focused on the bioactive compounds of red seaweed, as most studies have recognized red seaweed as a good source of protein and the current knowledge of the health benefits of red seaweed is largely based on in vitro and animal studies. Currently, regulations regarding the disclosure of mineral, heavy metal, and iodine content in seaweed products, as well as safe portion sizes, are insufficient. To ensure the safety and sustainability of seaweed as a food source, the industry should implement a comprehensive monitoring program for heavy metals and iodine or explore innovative processing methods to reduce harmful contaminants like arsenic.

Seaweed fibers, such as xylan, laminarin, and ulvan, have not received official EFSA approval and, thus, more research is needed to ascertain whether these carbohydrates are safe for human consumption. EFSA concluded that sodium alginate failed to reduce postprandial glycemic responses without a disproportionate increase in postprandial insulinemic responses and, thus, a health claim was rejected. Despite growing in vitro and in vivo evidence supporting fucoidan’s potential anti-obesity benefits, human research on this seaweed-derived compound remains limited. A single randomized double-blind, parallel, placebo-controlled trial found that overweight/obese individuals who consumed fucoidan for 3 months experienced a reduction in diastolic blood pressure and LDL cholesterol but no significant changes in weight, body composition, or other metabolic markers. The dose of fucoidan chosen for this study was 500 mg/d [112]. Animal studies suggest that low-molecular-weight fucoidan reduces LDL and cholesterol in blood but no studies have been conducted in humans to support its LDL-lowering effect. Despite existing regulatory guidelines, clearer regulations and guidance regarding permissible arsenic levels in food products are needed. The UK Food Standards Agency has warned against consuming Sargassum fusiform due to its high inorganic arsenic content. Regular environmental assessments and analysis of arsenic species in seaweed-containing foods are necessary to minimize health risks. Indirect arsenic exposure through livestock feed or fertilizer use should also be considered. While most edible seaweeds contain safe levels of heavy metals, ongoing monitoring and regulation are essential [113,114].

### Harmful Effect of Gold Algae

It is difficult to say that all seaweeds are salubrious for health, as golden algae such as *Prymnesium parvum* are not recommended for consumption, as they are found to be harmful to health in many research studies as they release toxins called Prymnesins. They have potent hemolytic, neurotoxic, and cytotoxic properties [115]. Large-scale fish mortalities have been observed globally [116]. *Prymnesium parvum* is often called golden algae due to its fucoxanthin pigments, which are found in the chloroplast of cells [117]. *Prymnesium* parvum produces a variety of toxins, including lipopolysaccharide-like compounds, proteolipids, galactoglycerolipids, and prymnesins. Prymnesins, first isolated and characterized by Igarashi et al. in 1995, exist in two forms: prymnesin-1 and prymnesin-2. Mass mortality in aquaculture ponds was noticed in gill-breathing animals in Poland and Germany in 2022 in the Odra River. Prymnesin causes cell swelling and cell lysis within a few minutes or it may take hours depending on the temperature or pH of the environment

Prymnesins are believed to interact directly with membrane components, particularly sterols, forming micelles that disrupt the plasma membrane. These detergent-like micelles create negatively charged pores, allowing cations to pass through exposed cells. Prymnesins can cause significant harm to fish by damaging their heart, liver, kidneys, nervous system, reproductive system, and endocrine system. Ultimately, these toxins can lead to death. While the direct effects of these toxins on humans are less pronounced, consuming fish contaminated with prymnesins can still be detrimental to human health.

While seaweed consumption in moderate amounts may offer potential benefits for weight management and overall health, further research is needed to establish its efficacy and safety. Long-term human clinical trials are essential for evaluating the potential risks and benefits of seaweed as a dietary supplement.

## 6. Conclusions

**Figure 5:**
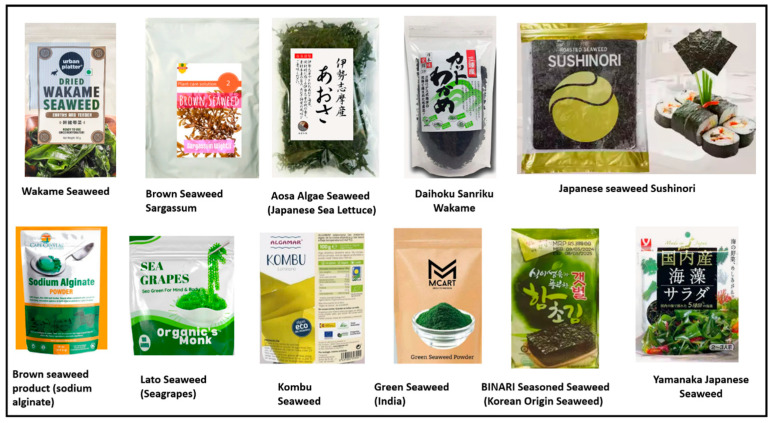
The commercial edible products of different kinds of seaweeds available easily in East Asia markets such as China, Japan, Korea, and India.

In many countries, seaweeds are being used as food additives and flavoring agents in the food industry. Various commercial products of seaweed are available in the Asian market, especially in China, Japan, Korea, and India (Figure 5). Japanese people have been using more than 88 seaweed species in meals and edible healthcare products. Among all seaweeds, three common seaweed products in Japan are nori, wakame, and kombu. Various studies have demonstrated that seaweeds are rich in minerals, essential fatty acids, protein, and fibers. Xanthigen, fucoxanthin, and punicic acid bioactive components of various seaweeds have the potential to suppress the differentiation of adipocyte cells and prevent the accumulation of triglyceride in adipocyte cells. Xanthigen downregulates the transcription factors and necessary proteins, which are useful for adipocyte differentiation.

The lipid content is lower in seaweeds relative to total dry weight. The total lipid percentage varies in different seasons in the same seaweed as, in *Saccharina latissimi*, the polyunsaturated fatty acid and total lipid are highest in March and November while lowest in January. The lipids obtained from seaweeds are highly digestible [114,118]. Humans cannot synthesize linoleic (18:2n-6) and α-linolenic acid (18:3n-3) and cannot convert α-linolenic acid into long-chain essential fatty acid but marine seaweeds have this limited ability to convert α-linolenic acid into eicosapentaenoic acid (EPA) and docosahexaenoic acid (DHA). Omega-3 and omega-6 are essential fatty acids that prevent the risk of cardiovascular disease, cancer, and mental illness and are responsible for the development of the nervous system [119,120,121].

This study was performed in red and brown seaweeds to see the potential source of fatty acid. The fatty acids were extracted from these seaweeds and analyzed by gas chromatography–mass spectrometry. The study reveals that *jubata* has the highest content of unsaturated fatty acids, including omega-6 fatty acids, while *C. jubata* and *U. pinnatifida* may be the seaweeds with the highest nutraceutical potential and have promising health benefits [12].

The animal experimental study showed that consumption of 1% *Undaria pinnatifida* lipid for 6 weeks decreased body weight significantly, while the consumption of *Undaria pinnatifida* lipid and n-3 PUFA-rich scallop phospholipids reduce the white adipose tissue content in a mice model [122]. Seaweeds have different concentrations of lipid content, with a maximum of 8.8% lipid present in *Porphyra* spp., while 74% total fatty acid is present in Plocamium Brasiliense [114,123]. Other seaweeds such as North Bornean seaweeds have also cardiovascular protective and neuroprotective effects [124] and may reduce fat content.

The seaweed effect for anti-obesity was also evaluated in human subjects. The red seaweed Gelidium elegans’ effect on obesity was observed in 78 subjects. Subjects were divided into groups: a placebo group (37 subjects) and 40 in an experiment group. People of 19–50 years old were included in the experiment. The BMI for inclusion criteria was 23.0 and 30.0 kg/m^2^. A 12-week regimen of daily Gelidium elegans extract (1000 mg) resulted in a reduction in both body weight and BMI. Although, LDL cholesterol and total cholesterol did not change significantly [125].

Existing research indicates a strong correlation between seaweed consumption and reduced obesity. In vitro studies employing 3T3-L1 cells exposed to seaweed extracts ranging from 6.25 to 100 µg/mL for preadipocytes and 1 to 10 µg/mL for mature adipocytes consistently demonstrated decreased triglyceride accumulation. Notably, a dose-dependent effect was observed in the majority of these experiments. Preclinical studies involving rats and mice have further supported these findings. Algae extract administered at dosages of 0.5% to 15% (*w*/*w*) in diet or 100 to 500 mg/kg body weight/day for periods of 4 to 16 weeks significantly reduced body fat accumulation in both visceral and subcutaneous adipose tissue in mice. Importantly, these effects were primarily observed in animals fed obesogenic diets. Seaweed extracts have demonstrated the potential to mitigate not only obesity but also associated metabolic disorders such as dyslipidemia, insulin resistance, and fatty liver disease. While the data are compelling for models of diet-induced obesity, the impact of seaweeds on weight management in individuals with established obesity remains less clear. Only a limited number of studies have investigated the effects of algae extracts in animals fed a standard diet. Furthermore, the efficacy of seaweed in counteracting genetic predispositions to obesity has been explored in only a few studies, such as those involving the red seaweed *Gelidium amansii* in mice.

Additional research is necessary to evaluate the therapeutic potential of seaweed extracts in treating established obesity.

The bioactive compounds of seaweeds play a crucial role in the management of obesity and weight management. However, the majority of investigations address the short-term effect of seaweeds or their ingredients on obesity in animal models. It shows a positive impact in obesity and weight reduction by increasing satiety and reducing energy intake. It also diminished nutrient absorption and delayed gastric clearance and the stimulation of stretch receptors in the stomach. However, chronic studies in humans are required to finalize the conclusion as an anti-obesity effect of seaweeds. Apart from this, the bioavailability of bioactive compounds of marine algae needs to be investigated in humans before considering it as a more potent anti-obesity therapeutic than pharmacological agents. The standard drugs for obesity and weight management have various side effects that can be reduced by utilizing natural seaweed and its bioactive ingredients. However, future investigation of therapeutic efficacy and mechanism of action of seaweeds is required.

Several potential mechanisms underlying the anti-obesity effects of seaweeds in preclinical studies have been proposed. Few studies suggest a reduction in food intake, while several studies support a reduction in nutrients in gut absorption. However, the most promising mechanisms involve alterations to metabolic pathways within target tissues and organs. Seaweeds have been shown to inhibit de novo lipogenesis, thereby reducing fatty acid availability for triglyceride synthesis in white adipose tissue. It is important to note that this metabolic pathway may be less prominent in human adipose tissue compared to rodent models. Ultimately, human clinical trials are essential to determine if the promising anti-obesity effects observed in animal studies can be replicated in people. This will help establish the potential of seaweeds as a preventative or therapeutic strategy for obesity management.

## Figures and Tables

**Table 1:** The different conventional drugs used for the treatment of obesity with mechanism of action.

Serial No.	Drug Class	Mechanism of Action	Examples
1	Stimulants	Increase norepinephrine and dopamine levels, increasing energy expenditure and suppressing appetite [91].	Phentermine
2	Lipase Inhibitors	Block the absorption of dietary fat by inhibiting pancreatic lipase [67].	Orlistat
3	Opioid antagonist	It blocks opioid receptors and produces the appetite-suppressing hormone proopiomelanocortin (POMC) [92].	Naltrexone
4	Antiepileptic drug	Topiramate is a medication primarily used to treat seizures and migraines. It also has the side effect of reducing appetite. While the precise mechanism is not fully understood, it is believed to involve the modulation of certain neurotransmitters, such as the inhibition of sodium channels and glutamate receptors [93].	Topiramate
5	Glucagon-Like Peptide-1 (GLP-1) Receptor Agonists	Mimic the effects of GLP-1, a hormone that promotes satiety and slows gastric emptying [94].	Liraglutide, semaglutide
6	MC4R agonist	It works on the lateral hypothalamic area to suppress the appetite [95].	Setmelanotide
7	Ghrelin Receptor Antagonists/Ghrelin Vaccine	Block the effects of ghrelin, a hormone that stimulates appetite [96].	Praltibetide (currently in development)
8	Combination drugs	Selectively inhibits the reuptake of dopamine and noradrenaline. In combination with Naltrexone medication, it increases feelings of fullness by stimulating the hypothalamus to produce more melanocyte-stimulating hormone (MSH), which helps reduce appetite and burn more calories [92].	Bupropion
